# Supplementary material for: Are carbon emissions trading and green financial instruments synergistic? -Comprehensive quantitative research based on content analysis
Source: PLoS One. 2024 Mar 7;19(3):e0298601. doi: 10.1371/journal.pone.0298601 (PMC10919601; doi:10.1371/journal.pone.0298601)
Supplement: S1 Data — (ZIP) [file pone.0298601.s001.zip › Data/地级市Z_F工作报告.zip!/╣π╢1⁄2/╔╟═╖╩╨/└┤╫╘╣1⁄2╓┌║┼í╛┬φ┐╦╩2╛▌═°í┐_2010.docx]

各位代表：

　　我代表市人民政府向大会报告工作，请予审议，并请市政协委员和其他列席人员提出意见。

　　一、2009年工作回顾

　　2009年，是我市积极应对国际金融危机，经济社会保持平稳较快发展的一年。在省委、省政府和市委的正确领导下，在市人民代表大会及其常务委员会和市政协的监督支持下，市政府以科学发展观为指导，按照省“三促进一保持”的工作部署，认真贯彻落实省粤东“两会”精神，迎难而上，奋力攻坚，经济社会发展取得新成绩，基本实现了“三年打基础”的阶段性目标。全年实现地区生产总值1035.73亿元，增长10.7%；地方财政一般预算收入58.54亿元，增长14.3%；全社会固定资产投资额291.9亿元，增长11.7%；社会消费品零售总额672.59亿元，增长18.2%；出口总额40.16亿美元，下降7.1%；中心城区居民人均可支配收入13651元，农村居民人均纯收入5260元，分别增长8.8%和7.7%。完成年度节能减排目标任务。

　　——产业结构调整取得新成效

　　工业经济优化发展。出台加快建设现代产业体系的决定和工业产业发展与布局规划。新增规模以上工业企业120家，完成规模以上工业总产值1533.66亿元，增长14.1%。实施品牌战略，新增中国驰名商标3件、省著名商标27件，省名牌产品18个，省出口名牌61个。获“中国文具生产基地”称号。大力推进产业转型升级和集群发展，企业自主创新能力不断提高。主导制定国家标准、省地方标准9项，新增高新技术企业24家、省级企业技术中心7家、专利授权3692件，实现高新技术产品产值403亿元，增长18.5%。玩具、服装等特色优势产业市场竞争力进一步增强。产业转移工业园获省认定为示范园区，成功竞得5亿元省产业转移扶持资金，深圳龙岗（汕头潮南）产业转移工业园首期基础配套设施基本完成。

　　服务业加快发展。第三产业增加值增长11.7%，对经济增长贡献率达43.1%，同比提高20.9个百分点，其中现代服务业完成增加值187.85亿元，增长6.1%。港口货物吞吐量突破3000万吨，汕头机场旅客吞吐量126.7万人次，增长16.1%。落实“家电、汽车、摩托车下乡”、“以旧换新”等扩大消费政策，城乡消费持续畅旺。推进生态滨海旅游示范区建设，实现旅游总收入74.29亿元，增长9.4%。蓝水星主题公园建成试业。金融机构本外币各项存款余额1623.06亿元，贷款余额570.47亿元，分别增长18.8%、33.7%。建筑业、房地产业健康发展。国家玩具质量监督检验中心建设顺利推进。玩博会、食博会、文博会等会展业加快发展。

　　农业现代化、产业化水平不断提高。完成农业总产值104.32亿元，增长4.2%。投入1700多万元，开展防旱抗旱工作，最大限度降低旱情损失。加快推进农田基本建设，加强良种良法示范推广。推进特色农产品基地和现代农业园区建设，牛田洋现代农业生态示范区被认定为“全国农产品加工创业基地”，潮南台湾农民创业园动工建设，澄海下岱美和潮南上南蔬菜现代化生产示范区完成首期建设，潮南“东华特绿”成为全市第一个农业集体商标。培育发展农业龙头企业和农民专业合作社，联结带动农民增收。稳步推进渔民转产转业。国家级海门中心渔港建设进展顺利，达濠一级渔港启动建设，云澳中心渔港初步设计通过国家农业部评审。加强农产品质量安全工作。强化动植物疫病防控。

　　——改革开放不断深化

　　中小企业发展壮大。出台促进中小企业健康发展政策措施，加大财税政策扶持，投入2.38亿元帮助中小企业有效应对金融危机。新登记私营企业2559户，增长22.8%，民营经济增加值占地区生产总值的60.6%。安排专项资金用于中小企业信用担保机构风险补偿，设立澄海兴信等4家小额贷款有限公司，发放贷款484笔2.08亿元。奥飞动漫、星辉车模、潮宏基成功上市,上市企业增至9家，万顺、黑牛、凯撒、皮宝四家企业通过证监会首发审核。

　　国有企业改革稳步推进。落实加快国有企业改革总体方案，13家优质企业改革重组进展顺利。全面完成澄海、潮阳、南澳县级供电企业管理体制改革。汕头商行获准实施重组，农信社、汕特农联社改革积极推进。

　　开放型经济持续发展。投入3000万元扶持外贸企业发展，引导提升产品质量，开拓新兴市场。实际吸收外商直接投资2.04亿美元，增长5.2%；外贸企稳回升，出口增幅居全省前茅。第106届广交会汕头企业成交总金额2.52亿美元，比上届增长9.8%。不断优化通关环境，“两港齐飞”战略取得新突破，广澳港区通过省一类口岸预验收并申报国家验收，新开通4条国际集装箱航线；汕头机场获准对东南亚国家和港澳地区航空公司飞机开放，开通至新加坡直航航线。保税区区位调整和申报综合保税区工作进展顺利。

　　区域合作进一步深化。主动融入海峡西岸经济区，积极争取国家政策支持，纳入《海峡西岸经济区发展规划》编制范围，成为海峡西岸旅游区核心城市之一。成功举办闽粤赣十三市党政领导第十四次联席会议暨2009年汕头招商引资经贸活动，强化与“海西”各城市的经贸交流合作。组团参加第二届粤东侨博会、粤东四市经济社会协作第三次党政联席会。

　　对台经贸合作取得历史性突破。打造粤台直接来往主通道，在全省率先实现对台海上货运直航，完成106航次1.76万标箱，实现对台旅游包船直航，市旅游总公司成为第二批指定经营大陆居民赴台旅游业务旅行社。率先建设粤台经贸合作试验区。

　　——重点基础设施建设全面推进

　　东部城市经济带项目取得突破性进展。与中国交通建设股份公司签订合作框架协议，新津、新溪、塔岗围片区相继启动建设。

　　重点交通项目加快推进。协调争取一批事关汕头当前和长远发展的重大交通基础设施项目，加快打通连接周边城市和经济腹地的快速通道，汕头至揭阳高速公路汕头段动工建设，汕头至揭西、揭阳至惠来、潮州至惠州高速公路汕头段前期工作强力推进。全力破解征地拆迁难题，完成厦深铁路汕头段征地工作，进汕头站联络线通过预可研评审。广梅汕铁路龙川至龙湖南段电气化改造、苏埃通道、阜鹰汕铁路、疏港铁路等重点项目前期工作进展顺利。南澳大桥加快建设。广澳港区防波堤工程完成前期工作，5万吨级航道基本完成主体工程，新临时避风塘建成并顺利搬迁，2号泊位基本建成，3号泊位复工建设。国道324线潮阳路段大修工程竣工开放交通，国道324线澄海路段、国道206线大学路段和省道237线改造工程前期工作加紧推进。

　　重点水利、能源项目有新进展。加快推进城乡防灾减灾水利工程，完成潮阳棉北海堤及南澳县城防工程达标加固、大龙溪一级水库除险加固工程，动工加固一批中小型病险水库，重建莲阳桥闸，完成西港桥闸重建、省人大议案机电排灌工程建设任务。南澳引韩供水工程前期工作基本完成，南澳过海电缆投入使用。华能海门电厂1、2号机组投入商业运作，完成500千伏送出工程建设。华润风电项目建成投产。松山电厂热电联产项目前期工作加快。

　　市政基础设施建设全面推进。强力推进金凤路桥征地拆迁工作，主体工程全线通车。金砂东路、海滨路、潮汕路、泰山路、汕樟北路（金平段）、黄山路、珠江路“水浸街”等一批道路工程和灯光亮化工程建成投入使用，海滨路西段改造工程进展顺利。第二过海水管顺利通水，实现南区双水源安全供水。排查整治公路危桥，磊口大桥、和平大桥动工改建。全力推进环保硬件设施建设，潮阳区污水厂一期工程投入运行，北轴、南澳污水厂投入试运行，峡山、两英污水厂主体工程完工，南区污水厂动工建设；泰山路截污干管工程完工，汕樟北路、海滨路等一批截污干管工程积极推进，粤东（汕头）环境监控与应急指挥中心建设进展顺利。旧城改造竣工新楼52幢34.68万平方米，回迁居民499户。

　　——经济社会管理日益强化

　　市场秩序更加规范。严厉查处制假售假、走私贩私、偷税逃税、商标专利侵权等经济违法案件，严厉打击欺行霸市行为，重点整治粤东水产中心市场。重视产品质量和食品安全，食品、药品、医疗器械监管机制不断强化。加强信息共享和经济户口管理，扎实推进纳税评估体系建设，规范行业税收征管，全面改进纳税服务，税收征管质量和效率不断提高。

　　节约集约用地试点工作初见成效。推进土地利用总体规划修编，核减耕地保有量指标18.82万亩。集中清理处置闲置土地50宗739.5亩，全面开展“三旧”改造工作。开发整理补充耕地1.18万亩，实现年度耕地占补平衡。启动问责制，严肃查处违法违规用地，整治工作通过国土资源部、省政府检查验收，获省节约集约用地奖。

　　社会治安、安全生产形势稳定。切实加强社会治安综合治理，深入推进“粤安09”、“创平安、迎国庆”重点打击行动、粤东四市联合打击行动等专项行动，严厉打击各类违法犯罪行为。开展“安全生产年”活动，推行镇街道安全生产委托执法，探索村居安全事故初始化自救应急救援试点工作，基层安全生产监管进一步加强，安全生产实现省政府下达的年度责任指标要求。重点整治省、市挂牌督办的火灾隐患重点地区，不断强化道路交通管理。海上渔业安全通讯救助网络全面建成，渔船救助能力大大提高。扎实推进全市应急体系建设，提高应急管理综合能力。

　　——社会各项事业协调发展

　　市容市貌明显改观。全面开展城市环境综合整治，整治集贸市场周边环境、创建样板路、拆除违法违章搭建物有新成效，海滨路亲水长廊部分开放使用。着力构建城市管理长效机制，强化“12319”城管热线协调督办作用，城市管理水平不断提高。加快海岸湿地国际示范区和林业生态建设。大力推进园林绿化工作，荣获“国家园林城市”称号。持续开展创建“六好”平安和谐社区，4个单位被民政部命名为“全国和谐社区建设示范街道（社区）”。

　　各类教育均衡协调发展。巩固提高义务教育，小学适龄入学率99.7%，初中毛入学率106.6%。加快普及高中阶段教育，新增学位约2万个，高中阶段毛入学率经省初步认定为70.2%。聿怀中学大洋校区建成交付使用，金中、侨中、鮀滨职中和澄海、潮阳职教中心等建设项目顺利推进。妥善解决中小学代课教师问题，录用“代转公”教师1069人。推进中小学校舍安全工程。投入7000万元，改善老区山区办学条件。

　　深化医药卫生体制改革。加强基层医疗卫生服务体系建设，促进基本公共卫生服务逐步均等化。提高农村群众医疗保障水平，新农合参合率97.1%。加强医疗急救网络建设，提高院前急救能力。落实重大传染疾病防控措施，做好甲型H1N1流感防治工作。

　　文化体育等事业繁荣发展。加快文化公共设施建设，市文化艺术学校新校区落成，市文化馆完成修缮并对外开放，潮剧艺术博物馆启动建设，新建农家书屋84家。创作潮剧《石榴花》、小型音乐剧《东方娃娃》等一批文化精品。编撰出版《汕头建筑》。完成文物普查田野调查工作，正式启动“南澳I号”水下考古抢救性发掘。积极开展非物质文化遗产保护工作，5个项目入选省级非物质文化遗产名录。大力开展全民健身运动，成功举办国际泳联10公里马拉松游泳世界杯等国内外大赛，汕头运动员获全运会金牌8枚。大力推进后进转化工作，人口与计划生育工作取得新成效，人口出生率9.92％。，自然增长率5.17％。。强力推进殡改工作，倡导殡仪新风。妇女儿童、双拥、老龄、慈善和社会福利事业加快发展，统计、人防、地震、气象、档案、方志、民族、宗教等工作取得新成绩。

　　就业形势保持稳定。落实积极就业政策，新增城镇就业岗位8.26万个，城镇登记失业率保持在3.2%以内。帮扶就业困难对象实现就业再就业5264人，职业技能培训3.5万人次，农村劳动力转移就业6.56万人。

　　社会保障体系不断完善。扎实推进社保扩面征缴工作。企业离退休人员月平均基本养老金增加173元，增长24%。妥善解决应保未保、早期离开国有集体企业人员社会保险问题。不断完善医疗和工伤失业生育保险制度，6万多名困难企业退休人员纳入医保范围，居民医保年度个人待遇增至10万元。低保扩面扎实开展，将年人均收入低于1500元的困难家庭全部纳入低保，超过4.3万户、10万人纳入低保。筹集廉租公房680套、经济适用房26套和租赁住房补贴830万元，为3000多户城镇低收入住房困难家庭提供保障。

　　一批群众关心的热点问题得到解决。加快农村饮水安全工程建设，基本解决26万多人饮水问题。规划停车泊位6655个，购置、更新公共汽车53辆，开通农村客运线路66条，建成农村客运候车亭220个，提前半年实现行政村通水泥路目标，群众行路难问题基本解决。完成中心城区45个“城中村”及涉农社区供水直抄到户改造3.5万户，13.8万人受益。落实扶贫开发“规划到户、责任到人”，推进48个老区山区扶持项目。扎实推进残疾人工作，建立健全精神病防治救助长效机制，解决482名贫困精神病患者、557名贫困白内障患者、73名贫困家庭先天性心脏病儿童、181名贫困家庭唇腭裂儿童治疗问题。

　　对口援建工作全面推进。投入1.18亿元援建草坡乡31个项目，29个项目开工建设，18个民生工程全面竣工交付使用。全力帮助草坡乡开展产业重建。发动海外侨胞助建“侨心居”220户。

　　——政府自身建设进一步加强

　　民主法制建设得到加强。出台加强市县政府依法行政意见，建立并实施市政府常务会议学法制度，依法行政制度建设取得新成效。办结人大代表议案（建议）86件、政协提案209件。提请市人大常委会审议法规草案2件，颁布政府规章4件、规范性文件20件，开展政府规章委托起草和政府规章后评估，政府立法质量进一步提高。开展行政处罚案卷评查和规范自由裁量权试点工作，实行重大行政决策法律审查制度，依法行政运行机制不断完善。全面推进政务公开，33个单位通过“民声热线”节目现场解答群众提问，畅通舆论监督渠道。积极开展网络问政，政府各级领导和专家学者在线听民声、解民困。落实信访工作责任制，开展领导干部大接访活动，妥善解决群众反映的热点难点问题，信访办结率达80%。

　　政府效能逐步提高。加快推进第四轮行政审批制度改革，开展行政审批提速专项工作，所有审批事项提速1/3以上。继续推进市行政服务中心建设，进驻单位56个927项业务，受理业务37万多件，办结率99.4%，即办件占75%。

　　财政支出结构进一步优化。深化市区县财政管理体制改革，加大对困难区县的扶持力度，推进基本公共服务均等化。落实党政机关厉行节约有关规定。加大民生投入，全市财政用于民生民安支出增加9.11亿元，占增支总额的68.5%。积极争取上级资金支持，获得中央扩大内需资金1.69亿元、中央代发地方政府债券3亿元，重点投入环保、水利、农业、教育、卫生等建设，成效明显。

　　廉政建设和行政监督成效明显。加强预算执行、重点投资项目、专项资金审计，积极稳妥开展经济责任审计。全面落实问责制，严厉打击违法违纪行为，规范权力运行。行政电子监察内容不断充实和拓展，45个单位428项行政审批事项纳入行政电子监察系统，实现省、市、区县和街道（镇）四级联网。强化责任意识，加强对重大事项、重大项目的督查督办，推动工作落实。

　　各位代表！过去一年，我市经济社会发展取得的成绩，是省委、省政府和市委正确领导的结果，是各级人大及其常委会和人民政协监督支持的结果，是全市人民同心同德、奋力拼搏的结果，也是社会各方面支持和帮助的结果。在此，我代表市人民政府，向全市人民，向中央和省驻汕单位，向驻汕人民解放军、武警官兵、人民警察和各民主党派、人民团体、社会各界人士表示崇高的敬意！向关心和支持汕头建设的港澳台同胞、海外侨胞和国际友人表示衷心的感谢！

　　我们也清醒地认识到，我市经济社会发展仍存在不少困难和问题：一是产业发展层次不高、结构不合理，主导产业分散，新兴产业、高新技术产业少，现代服务业特别是生产性服务业发展滞后，缺乏大项目尤其是大的先进制造业和现代服务业项目。二是经济开放度不高，招商引资和外贸总量小，与经济特区和著名侨乡的地位不相称。三是投资拉动仍然乏力，投资率低，生产性投资占比偏低，社会投资相对低迷，经济增长后劲不强。四是财政收支矛盾突出，税收收入占一般预算收入及地区生产总值比重偏低，可支配财力非常有限，政府偿债、落实扩大内需项目地方配套资金等刚性支出任务重，社会保障、公共服务等民生支出压力大。五是社会事业发展滞后，教育、卫生、社保、社会救助等水平仍然偏低，治安形势依然严峻，发展环境仍需改善。六是政府执行力与新形势、新任务的要求还不适应，服务意识、责任意识和落实意识亟待提高。对这些问题，我们必须高度重视，认真解决。

　　二、2010年经济社会发展主要目标和工作安排

　　2010年是实施“十一五”规划的最后一年，也是向“五年大变化”迈进的关键一年，更是机遇多于挑战的一年。我们既要看到，我国经济回升的基础还不牢固，经济形势较为复杂，更要看到，国内经济社会发展基本面长期向好，台海两岸关系继续积极发展，珠三角改革发展和海西经济区建设步伐加快，省委、省政府出台促进粤东地区实现“五年大变化”指导意见，省委十届六次全会明确提出汕头要在未来的粤东发展中独树一帜，为汕头加快发展注入新的动力。我市经济社会发展已步入良性发展轨道。我们要善于捕捉和把握难得的发展机遇，发现和培育有利因素，狠抓工作落实，千方百计推动汕头经济社会平稳较快发展。

　　今年政府工作的指导思想是，全面贯彻落实党的十七届四中全会、中央经济工作会议和省委十届六次全会、市委九届八次全会精神，以科学发展观为统领，以转变经济发展方式为核心，以自主创新为动力，围绕实现“五年大变化”、加快打造区域中心城市的目标，调整经济结构，深化改革开放，扩大投资和消费需求，大力实施现代服务业和先进制造业双轮驱动，着力提高基础服务和基本保障水平，真抓实干，狠抓落实，推动经济社会又好又快发展。

　　经济社会发展主要预期目标是：地区生产总值增长12%，单位生产总值能耗下降、二氧化硫和化学需氧量排放总量减排均实现“十一五”目标，全社会固定资产投资额增长18%，第三产业增加值占地区生产总值40.5%以上，地方财政一般预算收入增长14.5%，全社会研究与发展经费支出占地区生产总值0.55%，外贸出口总额增长8%，社会消费品零售总额增长18%，居民消费价格总水平涨幅控制在3%以内，城镇登记失业率控制在3.5%以内，城镇居民人均可支配收入增长9%，农村居民人均纯收入增长8%，人口自然增长率控制在7％。以内。

　　为实现上述目标，要着力做好七方面工作：

　　（一）优化提升产业结构

　　提升工业发展质量。做大工业经济堆头，加快发展石化深加工、能源和装备制造等大项目和支柱产业，积极培育文化创意、新材料和新能源等新兴战略性产业。实施名牌战略和技术标准战略，积极创建国家包装印刷名城和轻工机械、输配电设备等特色产业基地，发展壮大文具、动漫玩具和装备制造等一批拥有驰名商标、名牌产品的企业。不断提高自主创新能力，支持重点企业与高校、科研机构组建产学研一体的技术战略联盟实体，运用高新技术、先进适用技术改造提升传统产业。积极推进创建国家知识产权示范城市工作，争取进入国家知识产权示范城市行列。鼓励企业上市融资、增资扩产，做强做大。

　　推进产业集群升级发展。加快玩具礼品、潮式毛衫、输配电设备等产业集群升级示范区建设，促进产业集聚和产业链延伸。推动澄海玩具等集体商标注册，推进省市共建玩具礼品先进制造业基地。实施城市中心区“优二进三”，提升扩大高新区，加快南区产业集聚发展。

　　加快发展现代服务业。依托工艺玩具、纺织服装等优势产业，建设一批特色物流园区，发展现代物流业。大力发展现代信息服务业，积极推进数字汕头、无线城市建设。加快建设公共检测和创新服务平台。发展金融服务业，积极推进汕头商行重组和农信社改革，加快发展信用担保机构和小额贷款，开展农村政策性保险。大力发展海滨渡假休闲旅游、乡村旅游、特色旅游，建设生态滨海旅游示范区。办好玩博会、服装博览会、美食节、食博会等特色展会。

　　积极发展现代效益农业。巩固和稳定粮食生产。加强农业标准化工作，推广良种良法，优化种养结构。加快供销社改革发展，搞活农村产品流通。加快台湾农民创业园、台湾农产品交易中心、牛田洋现代农业生态示范区建设，启动生态经济带建设。加快建设海门中心渔港、达濠一级渔港、云澳中心渔港。推进农村信息化步伐。全面铺开集体林权制度改革。加强动植物防疫检疫，提高农产品质量安全水平。

　　（二）着力推进重大项目建设

　　加快东部城市经济带建设。全力推进新溪、塔岗围片区吹填和新津河口、外砂河口治理工程，尽快启动桥梁、滨海大道等配套工程。抓紧完成新溪、塔岗围片区控制性详细规划编制工作。妥善解决用地用海历史问题。着手澄海培隆围及六合围填海工程前期准备工作，逐步实现东部城市经济带向盐鸿方向延伸。

　　加快省示范性产业转移工业园建设。推动潮南产业转移园企业进园建设，加快推进疏港大道、濠江电子电路工业基地、南山湾产业园和潮阳海门石化产业园区基础设施建设，发挥省示范园带动作用。力争开工建设华能海门电厂3、4号机组。

　　加快基础设施建设。加快建设南澳大桥、汕头至揭阳、汕头至揭西、揭阳至惠来高速公路和厦深铁路汕头段及厦深铁路进汕头站联络线。抓紧苏埃通道、阜鹰汕铁路、疏港铁路前期工作。加快金凤路桥、国道324线澄海路段、国道324线潮阳路段、国道206线大学路段、和惠公路潮南路段首期6.35公里大修、陈沙公路延长线、两铜线潮南路段等工程建设改造步伐。加快广澳港区建设，开工建设防波堤工程、2万吨级石化码头，推进口岸查验“一站式服务”综合办公楼建设，完成一期工程和5万吨级航道建设，完成10万吨级集装箱码头前期工作，提升港区集疏运功能。积极推进海门港区粤东煤炭中转基地建设。完成莲阳桥闸重建主体工程，动工建设下埔桥闸改造达标加固工程。加快潮阳、南澳引韩供水工程建设。继续推进城乡水利防灾减灾工程和潮南上金溪等专项病险水库除险加固工程。

　　（三）不断提高开放水平

　　加快建设粤台经贸合作试验区。推进南山湾产业园、粤台商品交易物流中心和台湾农民创业园等载体建设，加强与台湾产业对接。积极争取建设南澳粤台两岸合作试验区。推动汕台货运直航健康发展，开展客货滚装业务。打好文化旅游品牌，促进粤台文化旅游交流。

　　加强区域合作。大力推进双转移，主动融入珠三角改革发展。充分利用闽粤赣十三市协作区、侨博会、粤东四市党政联席会议等各种平台，加强与粤东、海西区域城市的交流合作，推动粤东地区经济一体化，主动融入海峡西岸经济区。吸引港澳服务业来汕发展，主动融入粤港澳紧密合作。依托侨乡、潮商优势，进一步密切与东盟的合作。

　　进一步扩大开放。争取国家尽快批准建设综合保税区，推动保税区与广澳港区实施区港联动。优化对外贸易结构，加快加工贸易转型升级，推动外贸增长方式转变。实施“走出去”战略，积极组团参与各种大型经贸交流活动，扩大进出口。推动外向型建筑业加快发展。

　　加大招商引资力度。落实招商引资目标责任制，实施考核奖惩，动员全社会力量开展招商引资。采取网上招商、小分队招商等形式，增强招商引资针对性，扩大招商引资规模，提升招商引资质量。做好招商引资后续服务，提高签约项目履约率。承办好广东省第六届“山洽会”，签约引进一批项目。

　　（四）扎实推进精细化管理

　　加强经济运行监管。重视经济统计工作，加强经济运行监测，及时协调解决经济运行中出现的新问题。积极培育税源，强化税收征管，提高财政收入得益率。规范非税收入管理。扶持和发展行业协会。继续深入开展安全生产年活动，着力重点行业领域的安全治理，推进安全生产应急管理，加强安全科技保障，进一步减少各类事故总量。强化市场调控和监管，完善价格调节基金制度，大力加强产品质量监管和打假、打私、扶优工作。

　　加强人口计生管理。落实人口计生目标管理责任制，加强流动人口计生管理，加大基层基础设施投入，促进基层计生管理的规范化和标准化。巩固提高一类地区，转化提升三类地区，力争人口计生工作达到全省中上水平。认真开展第六次全国人口普查。落实殡葬管理目标责任制和目标考核制度，巩固和提高火化率，大力推进绿色殡葬。

　　扎实推进节能减排。严格落实节能减排工作责任制，强化节能减排刚性约束。继续推进重点领域和重点企业节能，推广节能技术和产品，推行清洁生产，加快淘汰落后产能。加快环保基础设施建设，完成南区、谷饶污水厂及配套管网建设，加快中心镇和练江沿线镇污水处理项目规划建设。积极推进汕头市雷打石环保电厂、澄海区洁源垃圾发电厂和潮阳区、潮南区、南澳县垃圾卫生填埋场建设。完成粤东（汕头）环境监控与应急指挥中心建设。

　　提高环境综合质量。加强饮用水源保护，继续开展生态示范创建活动。推进练江流域、莲花山尾矿环境污染整治。加快贵屿环保基础设施建设和环境综合整治，探索循环经济路子。扎实推进林业生态建设，强化河口、海岸湿地和近岸海域环境保护。

　　节约集约利用土地资源。强化土地执法监察，严格执行土地利用总体规划和土地用途管制，加强耕地特别是基本农田保护。加快盘活闲置和低效用地，推进土地经营储备。优化土地布局和用地结构，保障重点项目和民生项目用地。点面结合、全面推开旧城镇、旧厂房和旧村居改造工程，重点推进海滨路西段片区、西港片区、新兴片区、小公园历史风貌保护区和旧城西片区等一批旧城改造项目，拓展用地空间。

　　（五）加快宜居城乡规划建设

　　完善规划修编。做好“十二五”规划编制工作。完成城市发展总体规划和土地利用总体规划修编。参与《粤东城镇群协调发展规划》编制。加快修订《汕头经济特区城市规划条例》，理顺城乡规划管理体制，加快镇村规划编制。

　　加强城市建设和管理。全力实施汕樟北路（龙湖段）等一批重点市政道路改造建设项目。维修加固公路及市政危桥。完善第二过海水管工程配套设施建设。深入开展城市环境综合整治，继续完善12319城管热线建设，进一步健全城市管理长效机制，巩固发展国家卫生城市、国家环境保护模范城市、国家园林城市成果。大力发展城市公共交通。推进交通行政综合执法体制改革，重点整治交通秩序。加强社会治安综合治理，提高路面见警率，严厉打击各类违法犯罪活动，强化流动人口和出租屋管理，集中整治治安重点地区和突出问题，进一步增强人民群众的安全感。

　　加快农村城镇化步伐。加大农村基础设施投入，组织实施农田水利基本建设。全力推进“规划到户，责任到人”扶贫开发工作，继续落实扶持老区山区项目。加快新增中央投资公路和行政村通自然村公路建设，大力发展农村客运。积极推进农村改水改厕工作。

　　（六）更加注重民生民安工作

　　优先发展教育。推进义务教育均衡发展，全面加快普及高中阶段教育，实现教育经费“三个增长”。加快金山中学小石校区、鮀滨职校新校区、华侨中学买楼续建、市特殊学校、金平和潮阳职教中心等项目建设。启动潮南职业技术中心建设。进一步落实“代转公”、义务教育教师绩效工资、教师福利待遇“两相当”工作。继续推进中小学校舍安全工程。进一步办好汕头职业技术学院和市高级技工学校，支持办好汕头大学、粤东高级技工学校。重视学前教育和特殊教育。

　　完善医疗卫生服务。加强基层医疗卫生队伍建设，推进薄弱乡镇卫生院改造和县级医疗卫生机构建设。新农合参合率达98%以上。增强公共卫生服务能力，加强重大疾病防治，提升突发公共卫生事件预测预警和处置能力。启动实施基本药物制度试点工作。推进市中心医院门诊急诊综合楼改扩建工程，启动市卫生学校易地重建工程。

　　加快发展文化体育等各项事业。加强思想道德建设，加大基层文化建设力度，推进省文明城市创建活动。加快实施新华书店和广播电视网络重组。支持潮汕侨批申报国家档案遗产和世界记忆遗产。整治净化网吧、荧屏声屏等娱乐环境。积极备战省运会。定期举办国内外大型体育赛事和高水平的文化娱乐活动。加快推进市福利中心建设，扩大居家养老覆盖比例。推进妇女儿童、老龄、双拥、慈善、残疾人事业发展。

　　加强就业和社会保障工作。实施“扩大与促进就业”民心工程。发展壮大职业培训和技工教育。做好城镇职工社保关系异地转移工作，加大社会保险扩面征缴力度，努力实现应保尽保。力争城镇职工基本医疗保险参保率达到90%以上，城镇居民医保参保率达到80%。落实优抚优待政策，加大低保扩面力度，提高低保标准和补助水平，加强社会救助工作，切实保障特殊群体基本生活。

　　着力改善群众居住条件。加大保障性住房建设力度，实物安置一批城镇低收入住房困难家庭。争取6月底前基本完成中心城区商品房确权发证遗留问题处理工作。促进房地产市场健康稳定发展。做好公房危房安全工作。扩大住房公积金归集覆盖面。完成中心城区40个“城中村”和涉农社区供水直抄到户改造，加强住宅小区二次供水管理。继续解决农村饮水安全问题。

　　全面完成对口援建任务。加快推进援建项目工程建设及后续工作，强化监管，全力保证援建工程质量。帮助草坡乡加快经济社会发展。

　　（七）加快建设服务型政府

　　加强依法行政。自觉接受人大及其常委会的监督和政协的民主监督，及时、高效办理人大议案和政协提案。完善政府立法工作机制，全面落实行政执法责任制。健全重大决策的规则和程序，增强政府决策的科学性和可行性。

　　深化体制改革。实施政府机构改革，探索实行“大部门制”，健全部门之间的协调配合机制，提高政府运作效能。推动市、县区、镇（街道）放权简政，扩大镇级政府行政事务管理和处置权限。深化财政管理体制改革，进一步理顺市、县区、镇之间的财政分配关系，实现财权与事权相匹配。继续做好第四批行政审批制度改革，大幅减少行政审批项目，全面取消市级设定的非行政许可审批事项。提高行政服务中心事项入驻率和即办率，创新审批方式，实行并联审批试点，进一步完善行政电子监察系统建设，实现行政审批提速提效。

　　提高公共服务能力。增加财政对公共教育、公共卫生、公共文化体育、公共交通四项基础服务和对生活保障、住房保障、就业保障、医疗保障四项基本保障方面的投入，使基本公共服务加速覆盖广大居民。

　　狠抓工作落实。健全政府执行机制，强化问责制和重大项目责任制，优化执行运作程序，提高政府执行力。建设行政审批和公共服务综合信息平台，积极推进网上办事办公。加大行政监察和审计监督，规范权力运行，重点加强涉及公共利益事项的监督。加强公务员队伍建设。高度重视信访工作，妥善处置群体性、突发性事件。

　　各位代表，我市经济社会发展已呈现良好的态势，面临难得的历史机遇。我们肩负着时代赋予的神圣责任，肩负着人民的重托！让我们高举中国特色社会主义伟大旗帜，紧密团结在以胡锦涛同志为总书记的党中央周围，在市委的正确领导下，团结依靠全市人民，奋力拼搏，为实现“五年大变化”，加快打造区域中心城市而努力奋斗！
